# Early and midterm results of frozen elephant trunk operation with Evita open stent-graft in patients with Marfan syndrome: results of a multicentre study

**DOI:** 10.1186/s12872-022-02777-5

**Published:** 2022-07-26

**Authors:** Kazimierz Jan Widenka, Monika Kosiorowska, Heinz Jakob, Davide Pacini, Wolfgang Hemmer, Martin Grabenwoeger, Thanos Sioris, Anton Moritz, Konstantinos Tsagakis

**Affiliations:** 1 Department of Cardiac Surgery, University of Rzeszow Poland, 60 Lwowska Street 60, 35-301 Rzeszow, Poland; 2 Department of Thoracic and Cardiovascular Surgery, West German Heart and Vascular Center Essen, University Hospital of Essen, Essen, Germany; 3 Department of Cardiac Surgery, S. Orsola Hospital, University of Bologna, Bologna, Italy; 4 Department of Cardiac Surgery, Sana Cardiac Surgery Stuttgart GmbH, Stuttgart, Germany; 5 Department of Cardiovascular Surgery, Hospital Hietzing, Vienna, Austria; 6 Tampere University Hospital Heart Center, Tampere, Finland; 7 Department of Thoracic and Cardiovascular Surgery, University Hospital Frankfurt, Frankfurt, Germany

**Keywords:** Frozen elephant trunk, International E-vita Open Registry, Marfan syndrome

## Abstract

**Background:**

Endovascular treatment of patients with Marfan syndrome (MFS) is not recommended. Hybrid procedures such as frozen elephant trunk (FET), which combines stent-graft deployment with an integrated non-stented fabric graft for proximal grafting and suturing, have not been previously evaluated. The aim of this study was to assess the safety and feasibility of FET operation in patients with MFS.

**Methods:**

Patients enrolled in the International E-vita Open Registry (IEOR) who underwent FET procedure between January 2001 and February 2020 meeting Ghent criteria for MFS were included in the study. Early and midterm results were retrospectively analyzed. Preoperative, postoperative and follow-up computed tomography angiography scans were analysed.

**Results:**

We analyzed 37 patients [mean age 38 ± 11 years, 65% men]. Acute or chronic aortic dissection was present in 35 (95%) patients (14 and 21 patients respectively). Two (5%) patients had an aneurysm without dissection. Malperfusion syndrome was present in 4 patients. Twenty-nine (78%) patients had history of aortic surgical interventions. The 30-day and in-hospital mortality amounted to 8 and 14% respectively. False lumen exclusion was present in 73% in stented segment in last postoperative CT. The overall 5-year survival was 71% and freedom from reintervention downstream was 58% at 5 years. Of the nine patients who required reintervention for distal aortic disease, one patient died.

**Conclusions:**

FET operation for patients with MFS can be performed with acceptable mortality and morbidity. In long-term follow-up no reinterventions on the aortic arch were required. FET allows for easier second stage operations providing platform for surgical and endovascular reinterventions.

## Background

Marfan syndrome (MFS) is an inherited autosomal dominant multisystem disease, caused by mutations in the FBN1 gene encoding fibrillin-1 [1]with skeletal, ocular and cardiovascular abnormalities. The estimated prevalence of MFS ranges from 0.5 to 1 in 10,000 live newborns and equally affects males and females [2]. The average life expectancy of the patients with MFS has improved over the years by 25%, mainly due to aggressive prophylactic surgical treatment [3]. The major risk is associated with ascending aortic rupture and dissection, hence the 2010 American Heart Association, 2014 European Society of Cardiology and 2013 Japanese Circulation Society guidelines recommend prophylactic aortic replacement in all patients with ascending aortic diameter exceeding 4.0 – 5.0 cm, depending on risk factors [4–6]. The golden standard in the patients with MFS is therefore ascending aortic replacement, including aortic root replacement with or without aortic valve replacement. The indications for concomitant arch and descending aortic replacement are less clearly defined. In patients with MFS, the arch is usually not enlarged during the initial operation of ascending aortic replacement, however, one in every three aortic events occurring during follow-up involves the distal aorta, and previous ascending aortic replacement is associated with fourfold increased probability of dilatation of descending thoracic aorta [7]. The question of indications and safety of concomitant surgery on the aortic arch and proximal descending aorta in the patients with MFS remains unanswered. In recent years a novel frozen elephant trunk (FET) technique was developed to enable performing extensive surgery on ascending aorta and aortic arch as a one-stage procedure [8, 9], with a higher reported incidence of permanent neurological deficit [9], compared to classic elephant technique [10]. Even though stent-grafts are not recommended in the patients with connective tissue disorder [11], the procedures including FET are used, if no other option is available [12]. One of the advantages of FET technique is a stable proximal landing zone which facilitates later distal interventions.

To evaluate the results of FET in the patients with MFS, we analyzed data from the International E-vita Open Registry (IEOR).

## Methods

The patient’s selection was approved by the Institutional Review Board of the University of Essen, Germany.

### Study population

1049 patients who underwent the FET procedure between January 2005 and January 2017 were enrolled in the IEOR. The patients were operated in nine cardiac surgery centers (Leipzig, Germany; Essen, Germany; Bologna, Italy; Barcelona, Spain; Birmingham, UK; Tampere, Finland; Vienna, Austria; Stuttgart, Germany; Rzeszow, Poland). A total of 37 patients [mean age 38 ± 11 years, men 65%] meeting Ghent criteria for Marfan syndrome were included in the study [13]. Thirty-five (95%) patients were presented with acute or chronic aortic dissection, whereas 2 (5%) patients with dilatation of the aortic arch or proximal descending thoracic aorta. Of the 35 (95%) patients who had an aortic dissection, 10 (27%) had acute type A aortic dissection, 4 (11%) acute type B aortic dissection, 14 (38%) chronic type A aortic dissection and 7 (19%) chronic type B aortic dissection, according to Stanford Classification [5]. Among 29 (78%) patients who had previously undergone surgery on the thoracic aorta we recorded: four ascending aortic replacements, twenty Bentall de Bono procedures, three David valve sparing procedures, four descending aortic replacements and one thoracic endovascular aortic repair (TEVAR) in the descending thoracic aorta. In all 14 patients with acute aortic dissection the clinical status was recorded according to Penn classification (Table 1). This study was approved by the Local Ethics Committee. Informed consent was obtained from each subject.

**Table 1:** Patient’s characteristics

Parameters	N = 37
*Demographics*	
Age, years	37.6 ± 11.19
Male gender	24 (64.9)
*Aortic Disease*	
AAAD	10 (27.0)
ABAD	4 (10.8)
CAAD	14 (37.8)
CBAD	7 (18.9)
Aneurysm	2 (5.4)
*Previous Surgery*	
Overall	29 (78.4)
Ascending aortic replacement	4 (13.8)
Bentall de Bono Procedure	20 (69.0)
David valve sparing procedure	3 (10.3)
Descending aortic replacement	4 (13.8)
TEVAR	1 (3.4)
Previous aortic interventions per patient	1.1 ± 0.44
Emergency surgery < 24 h	12 (32)
*Penn Classification type A & B*	
Overall	N = 14
Class A	9 (64.3)
Class B	3 (21.4)
Class C	1 (7.1)
Class BC	1 (7.1)

Continuous variables are reported as mean and standard deviation; categorical variables are reported as percentages. Data are presented as number (%) unless otherwise indicated

*AAAD* acute type A aortic dissection; *ABAD* acute type B aortic dissection; *CAAD* chronic type A aortic dissection; *CBAD* chronic type B aortic dissection; *TEVAR* Thoracic Endovascular Aortic Repair

Penn Classification: Class A—Absence of branch vessel malperfusion or circulatory collapse, Class B—Branch vessel malperfusion with ischemia, Class C—Circulatory collapse with or without cardiac involvement, Class BC—Branch vessel malperfusion and circulatory collapse

### Patient management

The anatomy of the aortic dissection and organ perfusion were carefully assessed in all patients radiologically and clinically on admission. The operative procedure was performed within 24 h in 12 (32%) patients. All patients were treated with the E-vita Open stent-graft (Jotec, Hechingen, Germany). Surgical technique and indications were as previously described [9]. No standard surgical protocol for aortic arch surgery or stent-graft placement and implantation was applied. All operations were performed through median sternotomy. Arterial cannulation was performed via the right subclavian artery in 28 (76%) of patients, the ascending aorta in 4 (11%), the right subclavian and femoral artery in 1 (3%) patient. Antegrade selective cerebral perfusion (10–15 ml/kg/min, 18–22 °C) was implemented in all patients (bilateral in 95% and unilateral in 5%). The use of a guidewire and the cerebrospinal fluid drainage was advocated, but not compulsory during FET implantation. The distal anastomosis was performed in Zone 3 in 28 (76%) of patients, Zone 2 in 8 (22%) and Zone 1 in 1 (3%) of the patients according to classification proposed by Ishimaru [14]. All three aortic arch vessels were implanted as Carell patch in 22 (60%) patients, brachiocephalic trunk and left common carotid artery as Carell with the separate left subclavian artery in 3 (8%) and all three arch vessels separately in 12 (32%) patients.

### CTA follow up

Aortic diameters, endoleaks, dissection extension, thrombosis or patency of true and false lumen and condition of the aorta distal to implanted hybrid stent-graft were analyzed by means of electrocardiography gated computed tomography angiography (CTA) or magnetic resonance imaging. All the measurements were taken in multiplanar reconstruction always in a plane perpendicular to the manually corrected local aortic centreline. Patients were followed up before discharge, at 6 and 12 months postoperatively, and annually thereafter, according to the standard protocol of the IEOR. Fate of aortic false lumen was assessed with the first and the last postoperative CTA scans. The mean follow-up of the discharged patients was 3.27 ± 2.94 years (95% CI 2.20–4.33, range 0.04–12.0 years) and was complete.

### Statistical analysis

Statistical analysis was performed using SPSS version 18.0 (SPSS, Chicago, IL). Continuous variables were reported as mean ± standard deviation, categorical variables were expressed as percentages throughout the report. Standard Kaplan–Meier actuarial techniques were used to analyze the cumulative survival curve for long term follow-up and freedom from aortic reintervention.

## Results

### Operative data

The average size of the Evita open was 26.4 ± 4.8 mm (range 20-40 mm). The aortic root was replaced during the FET procedure in 10 patients (27%) and ascending aorta in 27 patients (73%). A total of 23 (62%) patients had the aortic root replaced during the first operation on the ascending aorta. In addition to FET, one patient underwent coronary artery bypass grafting and four patients underwent mitral valve surgery (three repairs, and one replacement). Cardiopulmonary, crossclamp, selective antegrade perfusion and visceral ischemia times were as follows: 254 ± 79 min, 154 ± 54 min, 73 ± 29 min, 67 ± 26 min. The operative data are summarized in Table 2.

**Table 2:** Operative data

Parameters	N = 37
*Arterial cannulation*	
Subclavian artery	28 (75.7)
Ascending aorta	4 (10.8)
Subclavian artery + femoral artery	1 (2.7)
Other	4 (10.8)
*Cerebral perfusion*	
Bilateral	35 (94.6)
Unilateral	2 (5.4)
*Proximal landing zone (Ishimaru)*	
Zone 1	1 (2.7)
Zone 2	8 (21.6)
Zone 3	28 (75.7)
*Aortic arch vessels reimplatnation*	
BCT + LCCA + LSA (Carell patch)	22 (60)
BCT + LCCA (Carell patch) + LSA separate	3 (8.1)
BCT/LCCA/LSA separate	12 (32.4)
ET (E-vita open) diameter (mm)	26.4 ± 4.8 (range 20–40)
*Concomitant procedure*	
AAR	27 (73.0)
Bentall de Bono	7 (18.9)
Aortic valve reconstruction (David)	3 (8.1)
AVR	1 (2.7)
CABG	1 (2.7)
MV Repair	3 (8.1)
MV Replacement	1 (2.7)
*Operative times (min)*	
CPB	254 ± 79
Cross clamp	154 ± 54
SACP	73 ± 29
Visceral ischemia	67 ± 26

Continuous variables are reported as mean and standard deviation; categorical variables are reported as percentages. Data are presented as number (%) unless otherwise indicated

*BCT* brachiocephalic trunk; *LCCA* left common carotid artery; *LSA* left subclavian artery; *FET* frozen elephant trunk; *AAR* ascending aortic replacement; *AVR* aortic valve replacement; *CABG* coronary artery bypass grafting; *MV* mitral valve; *CPB* cardiopulmonary bypass; *SACP* selective antegrade cerebral perfusion

### Early results

Overall, 3 (8%) patients died within 30-days after FET. Five (14%) out of 37 patients died in the perioperative period. In-hospital mortality was due to multiorgan failure in four patients and left ventricular failure in one patient followed by left ventricular assist device implantation. Among the in-hospital deaths, three patients had previously undergone aortic operations: Bentall de Bono procedure or ascending aortic replacement and one patient had been subjected to Bentall de Bono procedure and TEVAR. A total of 7 (19%) patients underwent rethoracotomy for bleeding. Neurological events occurred in four (11%) patients. Postoperative cerebral complications included transient neurologic dysfunction in 2 (5%) patients. The overall 3 (8%) patients sustained spinal-cord injury (SCI): two patients with the injury being complete (paraplegia) and a patient with incomplete one (paraparesis). The incidence of permanent neurological deficit was 5% and was associated to spinal cord injury. One patient experienced both transient stroke and paraparesis. Nine (24%) patients required ventilation for more than 72 h. Acute renal failure requiring dialysis occurred in 11 patients (30%), of which 9 (24%) patients were subjected to temporary dialysis, while 2 (5%) required permanent dialysis (Table 3).

**Table 3:** Outcomes

Outcomes	N = 37
Exploration for bleeding	7 (18.9)
*Neurological events*	4 (10.8)
Transient neurologic dysfunction	2 (5.4)
Permanent neurologic dysfunction	2 (5.4)
*Spinal-cord injury*	3 (8.1)
Paraplegia	2 (5.4)
Paraparesis	1 (2.7)
Prolonged ventilation time (> 72 h)	9 (24.3)
*Renal failure requiring dialysis*	11 (29.7)
Temporary dialysis	9 (24.3)
Permanent dialysis	2 (5.4)
*Endoleak*	
Ia	0
Ib	1 (2.7)
II	1 (2.7)
In-hospital mortality	5 (13.1)
30-day mortality	3 (8.1)
*Aortic re-interventions*	
Overall	9 (24.3)
TEVAR	3 (8.1)
Open surgery	6 (16.2)

Categorical variables are indicated as counts and percentages

*ICU* Intensive Care Unit; *TEVAR* Thoracic Endovascular Aortic Repair

### CTA follow-up

Postoperative CT was available in 36 out of 37 operated patients. At the level of the implanted FET complete or partial thrombosis of the false lumen (FL) of the aorta was achieved in 30 (88%) patients; of which FL thrombosis was complete in the first postoperative CT in 17 (50%) patients, and in 13 (38%) partial. In the last performed angio CT, complete or partial thrombosis was achieved in 28 (93%) patients; including 22 (73%) with complete and 6 (20%) with partial thrombosis. Patent FL was present in four (12%) patients and two (7%) patients at the first and last postoperative CT scans respectively. The rate of the FL patency, distal to the stent-graft was higher; 47% (16) and 37% (11) at the first and the last postoperative CT scans respectively. The complete FL thrombosis distal the FET was observed in six (18%) and five (17%) patients on early and late postoperative CT scans respectively (Table 4). In both patients with thoracic abdominal aneurysm, aneurysms remained excluded during the follow-up period.

**Table 4:** Fate of aortic false lumen as assessed by computed tomography

	First postoperative CT scan (N = 34)	Last postoperative CT scan (N = 30)
*Stent-graft level*		
FL thrombosis	17 (50.0)	22 (73.3)
FL partial thrombosis	13 (38.2)	6 (20.0)
FL patent	4 (11.8)	2 (6.7)
*Distal to stent-graft*		
FL thrombosis	6 (17.6)	5 (16.7)
FL partial thrombosis	12 (35.3)	14 (46.7)
FL patent	16 (47.1)	11 (36.7)

Categorical variables are indicated as counts and percentages

*FL* false lumen; *TAA* thoracic aortic aneurysm; *CT* computer tomography

### Late follow-up

**Fig. 1:**
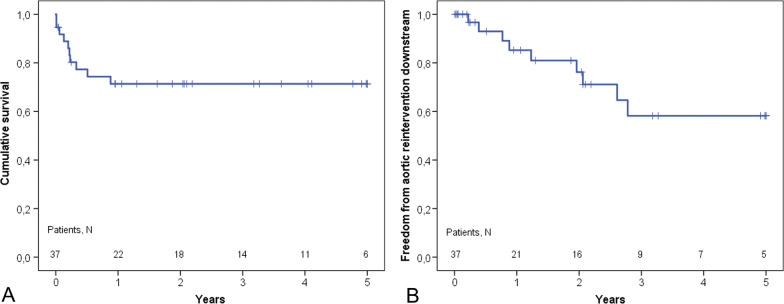
Cumulative survival (**A**) and freedom from aortic reinterventions downstream (**B**) in patients with marfan syndrome and frozen elephant trunk procedure

**Fig. 2:**
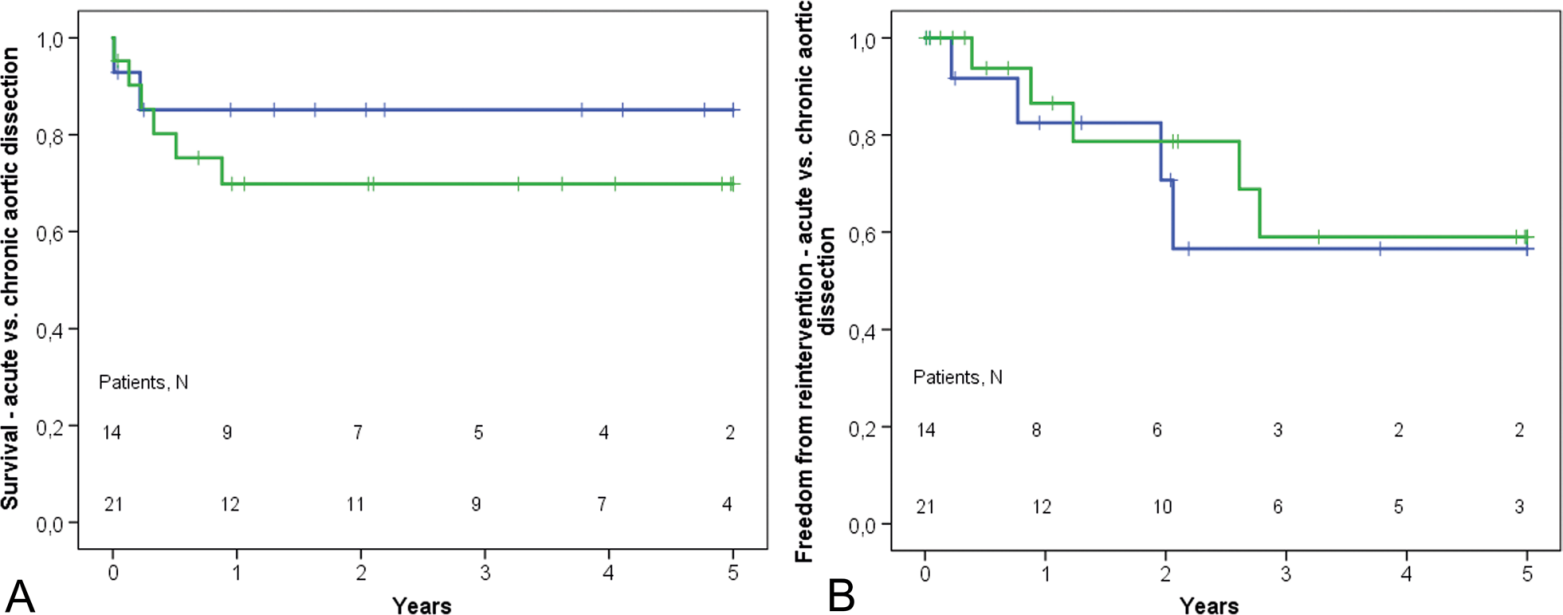
Cumulative survival (**A**) and freedom from aortic reinterventions downstream (**B**) in patients with marfan syndrome and frozen elephant trunk procedure—acute vs. chronic dissection

The actuarial survival at five years was 71.3% (Fig. 1). Nine patients required reintervention on the descending thoracic aorta, three underwent TEVAR and six open surgical repairs. The actuarial freedom from reintervention downstream was 58.2% at five years (Fig. 1). There was no statistical difference in patients with acute and chronic aortic dissection in terms of survival and reoperation rates (Fig. 2).

## Discussion

The long-term survival of patients with Marfan syndrome has improved over the years [3, 15]. The five-year survival of patients with MFS following prophylactic and emergency surgery is 97% and 51% respectively [15]. The improvement is therefore mainly attributed to the aggressive prophylactic replacement of the ascending aorta [3, 15]. The remaining aorta, however, becomes the major cause of morbidity and mortality [7, 15–17]. In patients with MFS, every third aortic event occurring during follow-up involves the distal aorta [7], and previous ascending aortic replacements are associated with a fourfold increased probability of dilatation of the descending thoracic aorta [7]. As shown by Hartog et al., the risk factors for distal aortic events in the patients with MFS include previous prophylactic of the ascending aortic surgery and the size of the descending aorta greater than 27mm [16]. The cut-off value is significantly lower than recommended in the guidelines. Due to the increased mortality and morbidity in for aortic arch replacement, the extent of aortic resection of the aorta is not equivocally advocated [18–20]. In the present study the aortic arch was replaced not on a prophylactic basis, but when indications existed, being either the aortic arch diameter, growth or arch dissection together with proximal descending aortic enlargement or rapid growth (> 10 mm per year) and aortic tear in acute dissection. The reported in-hospital mortality of 13% is similar to previously published series of aortic arch replacement in MFS patients, including reoperations [20–23]. Ma Wei-Guo et al. in the recently published study showed significantly better results, with an operative mortality of 6.6%, in patients with MFS and FET implantation for chronic and acute aortic dissection [24]. However, in our series, 78% of the patients underwent reoperation, as opposed to 21.7% in the Ma Wei-Guo et al. The mortality in our series is mainly related to reoperations (17% vs 7%). Mortality in FET operations depends on the patient selection as shown by Tian et al., who in meta-analysis presented mortality of 0 to 18.2% depending on the publication [25].

The use of FET and TEVAR in the patients with connective tissue disorder is not recommended in elective situations [4, 5], but it is not uncommon in emergency procedures and as a rescue if a safe distal anastomosis cannot be performed in acute dissection [19, 25]. In our series only one patient experienced a FET-related distal aortic injury on the first postoperative day, which eventually expired. There were no late complications related to FET. Despite the risk and unknown fate of FET in the patients with MFS, large series of patients are reported in the literature [19, 24–26], with satisfactory early and late outcomes. In the published meta-analyses by Tian et al. FET in the patients with Marfan was used in ten series and accounted for 8.6% of the operated patients (range, 2.4–21.2%) in 10 studies [25]. The use of TEVAR, despite the existing recommendation, is even more common. Searching for “TEVAR and Marfan Syndrome” in the ScienceDirect database resulted in 315 publications, clearly showing that clinical practice differs from the guidelines. The reported results of TEVAR are without doubt suboptimal [27–30]. The endoleak rate is substantial in the patients with MFS, and in the meta-analysis performed by Pacini et al. it occurred in 22% cases (16% type I, 4% type II, and 2% type III), with low operative mortality in 1.9% and substantial 2.5-year mortality of 13% [29]. There are two new iatrogenic complications of TEVAR: stent-graft induced new entry (SINE) and retrograde type A dissection, both are serious device-related complications occurring after TEVAR regardless of etiology, but are more common in the patients with connective tissue disorders [27, 29]. In the patients undergoing FET, the risk of stent-graft related complications is limited by the proximal surgical fixation. Therefore, there is no risk of retrograde aortic dissection. In our series the incidence of SINE was limited to one patient, as was in Ma et al. series [24]. One of the reasons for the lower rate of SINE in FET, may be the issue of stent-graft oversizing, which is obligatory in TEVAR. It has previously been shown to be the risk factors for SINE in TEVAR [28]. In FET procedure the oversizing is not necessary.

The stented portion of the EVITA Open Plus provides a stable platform for the subsequent implantation of a distal aortic stent-graft if needed. Pacini et al. showed that there was no endoleak in previously implanted stent-grafts in patients with MFS, in contrast to the landing zone in the native aorta, where endoleaks were found in 29% of the cases [29]. Bleeding during open surgical procedures for the distal aorta can be controlled with standard balloons, used for transcatheter aortic valve implantation, in order to avoid cross-clamping of the stent-graft. The technique was used in one of the patients operated on in our series and also reported by Ma et al. [24].

### Neurological complications

One of the serious complications following FET are permanent and transient neurological deficits [24–27, 31, 32],. The data is confusing as they come from small series and retrospective studies [32]. In a meta-analysis of 50 publications on hybrid aortic arch procedures, the incidence of permanent or transient stroke ranged from 1.0 to 16.0%, with a pooled event ratio of 6.2% for FET, 0.8% to 18.8% in the debranching group (pooled ratio 7.3%) and 1.6% to 25.0% in the stented elephant trunk group (pooled ratio 10.9%) [32]. In our series of patients with complications, the stroke occurred in 5% of the patients and was transient. The results are in line with to the reported stroke rate for classic surgical arch replacement [10], including patients with acute ascending dissection following hemiarch replacement, as reported by Rylski et al [21].

SCI following FET is considered a relatively ‘new’ complication in open aortic arch surgery [31]. Yet, it is not the case, as it was reported previously, and related both to an ET graft longer than 8–10 cm in the descending aorta and to clotting around the graft with a risk of peripheral thrombo-embolism [33, 34]. The risk of spinal cord injury following FET is however higher than following classic aortic arch replacement. Potential causes of spinal cord damage during the operation include: duration of circulatory arrest, core body temperature during the circulatory arrest, coverage of segmental arteries by stent-graft implantation, embolism and postoperative hemodynamic management [31]. In the meta-analysis by Cao et al., it was higher than in the case of debranching and stented ET and ranged from 1.3 to 25.0% (pooled ratio 7.9%) [32]. In the present study the risk of SCI was 8%, in 2 patients it was permanent while in 1-transient. As the protection of the spinal cord improves, the result will also improve, as shown by Ma et al., who reported the surprisingly low rate of SCI of 0.9% in 106 patients operated for acute aortic dissection in patients with MFS and FET [24].

### Long term clinical and angiographic results

Patent FL is a major risk factor for the need for reintervention on the aortic arch and distal aorta in patients after repaired Type A dissection and MFS [18, 19, 21, 22, 35]. The FET procedure as previously reported, allows a high rate of FL partial or complete thrombosis and may result in fever rate of reoperations on distal aorta [12, 36, 38, 39]. In our series of 37 patients with MFS the rate of FL thrombosis was high with only 7% patency at the level of the stent-graft and 37% distal to the stent-graft (Table 4). However, nine patients required reintervention on the distal aorta (TEVAR in 3 patients, open surgery in 6 patients), resulting in 58.2% actuarial freedom from reintervention downstream at five years. In Ma et al. series of patients with MFS the FL remained patent in 10.8% of the patients at 2 years after the initial operation [24], and freedom from reoperation at 5 years was 88.8% (95% CI, 80.08–93.89%) [24]. In the present study, the five-year actuarial survival was 71.3%. There was no difference between patients with acute and chronic aortic dissection in terms of long-term survival (Fig. 2), as previously shown by Ma et al [24].

### Study limitations

The current study has several limitations. Firstly, this is a retrospective and non-randomized study based on the IEOR. Secondly, because of the multicenter design of the study, different surgical protocols were followed, which were not standardized across the different centers. Thirdly, the genetic and/or histologic confirmation of MFS was necessary but Ghent criteria were used to identify patients with MFS. Genetic screening of MFS is not routinely used and the diagnosis is made according to the clinical criteria of the Ghent nosology with 95% accuracy [37]. This study is a real-life representation of surgical practice and results. As opposed to single-site publications, there is no bias of centre of excellence good results, that are impossible to reproduce in large registries of similar procedures.

## Conclusions

Although FET operation for the patients with MFS with extensive aortic arch pathology seems controversial, it may be performed with acceptable mortality and morbidity, as shown in this high-risk series. FET procedure allows a high rate of FL partial or complete thrombosis in stent graft level and distally and may result in reduced the number of secondary interventions on downstream aorta. FET allows for easier second-stage operations, providing a platform for both surgical and intervascular reinterventions. Further follow-up is mandatory to assess long-term results of FET in the patients with MFS.

## Data Availability

The datasets used and/or analysed during the current study are available from the corresponding author on reasonable request.

## Abbreviations

CT: Computed tomography
FET: Frozen elephant trunk
FL: False lumen
IEOR: International E-vita Open Registry
MFS: Marfan syndrome
SINE: Stent-graft induced new entry
SCI: Spinal cord injury
TEVAR: Thoracic endovascular aortic repair
